# Ionic Liquids: evidence of the viscosity scale-dependence

**DOI:** 10.1038/s41598-017-02396-7

**Published:** 2017-05-22

**Authors:** Quentin Berrod, Filippo Ferdeghini, Jean-Marc Zanotti, Patrick Judeinstein, Didier Lairez, Victoria García Sakai, Orsolya Czakkel, Peter Fouquet, Doru Constantin

**Affiliations:** 1 Laboratoire Léon Brillouin, CEA, CNRS, Université Paris-Saclay, CEA Saclay, 91191 Gif-sur-Yvette, Cedex, France; 2 Lawrence Berkeley National Laboratory, Energy Storage Group, 1 Cyclotron Road, Berkeley, CA 94720 USA; 3 Laboratoire des Interfaces Complexes et de l’Organisation Nanométrique, ECE-Paris Ecole d’Ingénieurs, 37 Quai de Grenelle, 75015 Paris, France; 4 Laboratoire des Solides Irradiés, École Polytechnique, CEA, CNRS, Université Paris-Saclay, 91128 Palaiseau, Cedex France; 5 ISIS Neutron and Muon Facility, Rutherford Appleton Laboratory, Chilton, Didcot OX11 0QX UK; 6 Institut Laue Langevin, 38042 Grenoble, Cedex France; 7 Laboratoire de Physique des Solides, CNRS, Univ. Paris-Sud, Université Paris-Saclay, 91405 Orsay, Cedex France

## Abstract

Ionic Liquids (ILs) are a specific class of molecular electrolytes characterized by the total absence of co-solvent. Due to their remarkable chemical and electrochemical stability, they are prime candidates for the development of safe and sustainable energy storage systems. The competition between electrostatic and van der Waals interactions leads to a property original for pure liquids: they self-organize in fluctuating nanometric aggregates. So far, this transient structuration has escaped to direct clear-cut experimental assessment. Here, we focus on a imidazolium based IL and use particle-probe rheology to *(i)* catch this phenomenon and *(ii)* highlight an unexpected consequence: the self-diffusion coefficient of the cation shows a one order of magnitude difference depending whether it is inferred at the nanometric or at the microscopic scale. As this quantity partly drives the ionic conductivity, such a peculiar property represents a strong limiting factor to the performances of ILs-based batteries.

## Introduction

As they show low flammability and electrochemical reactivity, Ionic Liquids (ILs) have been identified as promising electrolytes for batteries^1^. The combination of a large diversity of anions and cations leads to a flourishing variety of ILs^2^, but they all share a common ground: the presence in their diffraction patterns of a so-called prepeak in the 0.2–0.5 Å^−1^ region. This prepeak is the signature of a semi-local (few nm) segregation^3^ in the liquid. Molecular Dynamics simulations (MD) suggest that this nano-structuration extends up to a scale one order of magnitude larger^4^ (few tens of nm). But, while such characteristic size should be easily detectable by Small Angle Neutron (SANS) or X-Ray (SAXS) Scattering techniques, no SANS or SAXS data report any nanometric spatial organization of pure bulk ionic liquids. A possible explanation is that the local evanescent organization responsible for the prepeak does not induce large enough density fluctuations able to create a contrast between the segregated regions of the liquid.

The ILs structuration has nevertheless been inferred, but rather indirectly, through the assessment of dynamical quantities by Quasi-Elastic Neutron Scattering (QENS). It has for example been shown^5, 6^, that at the molecular scale and short time (few tens of ps) a confinement volume could account for a localization of ILs molecules within nanometric aggregates. But, as QENS probes the system on a local scale (few nm at most) the expected few tens of nm characteristic size of the nanometric structuration remains unreachable.

In the present paper, we ally Time-of-Flight (ToF) QENS, Neutron Spin Echo (NSE), Dynamical Light Scattering based particle-probe rheology (DLS-PBR) and Pulsed Field Gradient Nuclear Magnetic Resonance (PFG-NMR) to reach a coherent structural and dynamical multi-scale view of the ILs physics, from the molecular to the microscopic scales. We first evidence that the transport properties of ILs are scale dependent. Then, we show that DLS-PBR is able to bridge both in space and time the process leading to such a scale dependence of the transport properties. All the experimental approaches used here, probe the spatial dependence of the dynamical modes through a common quantity: Q, the scattering vector (proportional to the inverse of a distance). As it is closely related to the ionic conductivity by the Nernst-Einstein relation, we are interested in a single quantity: the translational diffusion coefficient of the IL. We focus our study on a canonical imidazolium based IL: 1-butyl-3-methyl imidazolium bis(trifluoromethanesulfonyl imide (BMIM-TFSI). The neutron scattering cross-section of protons being much larger than the ones of all the isotopes in BMIM-TFSI, QENS and NSE are only sensitive to the self-correlation dynamics of the BMIM cation. We also study a specifically deuterated BMIM cation, in order to validate the neutron model of the cation dynamics we propose.

## Results

### Neutron scattering: probing the cation CoM long-range diffusion at the molecular scale (ps-ns/0.1–10 nm)

**Figure 1:**
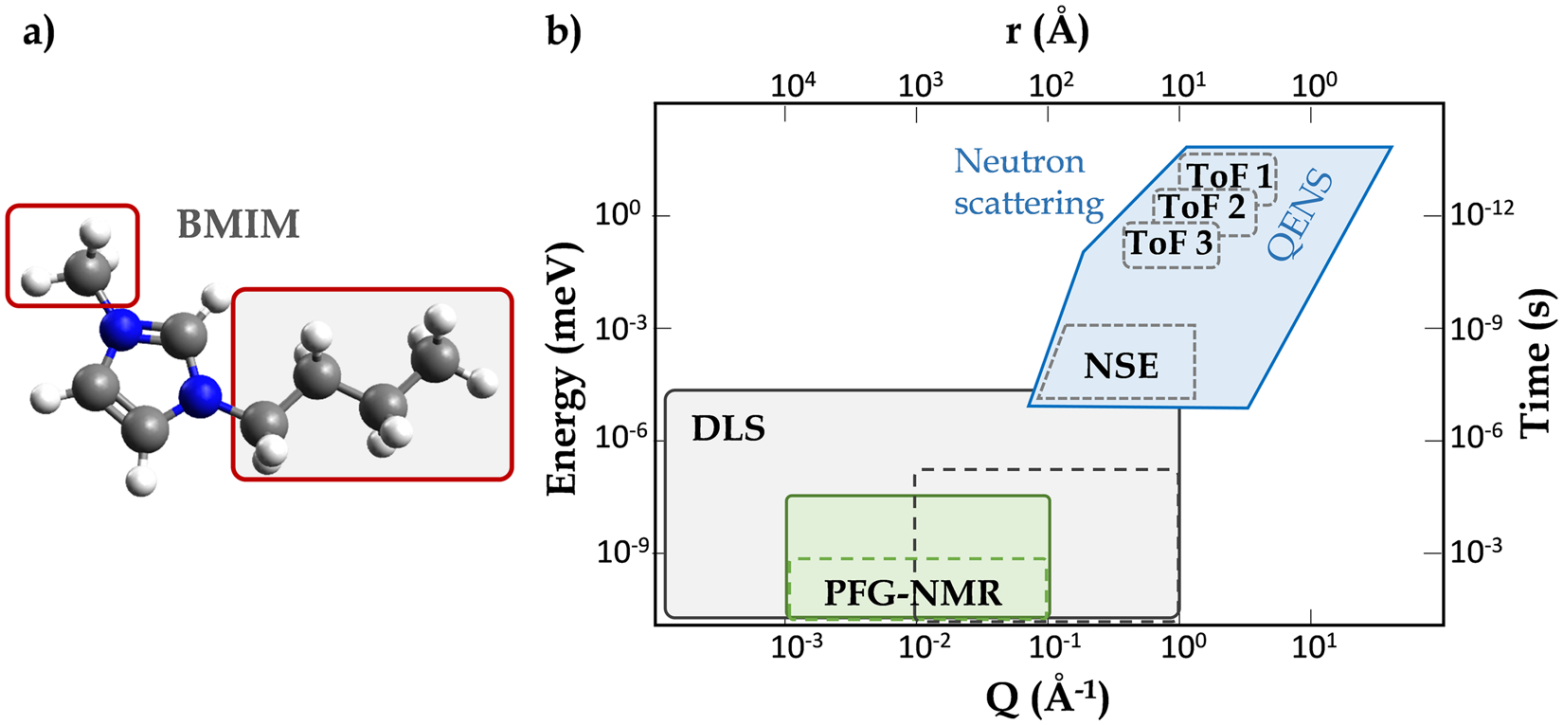
(**a**) This study focuses on the multi-scale dynamics of the cation of BMIM-TFSI. On the chemical structure above the red squares identify the methyl and alkyl side-chains carried by the imidazolium ring. While the quantity of interest here is the long-range translational diffusion coefficient of the center-of-mass of the whole cation, the QENS/NSE signal is very sensitive to the dynamics of the side-chains. The neutron experiments have been carried out on the fully protonated cation, but in order to challenge the robustness of the model we propose, the neutron contribution of the alkyl side-chain (squares filled in grey color) has been hidden by deuteration. Throughout this paper, this selectively deuterated cation sample will be called B(d9)MIM-TFSI. **(b)** Space and time plot illustrating the scales probed by Time-of-Flight (ToF) Quasi-Elastic Neutron Scattering (QENS), Neutron Spin-Echo (NSE), Pulsed-Field Gradient Nuclear Magnetic Resonance (PFG-NMR) and Dynamic Light Scattering (DLS). The shaded lines indicate the generic space and time scales accessible with these techniques. The scales specifically accessed in the present paper are shown with dotted rectangles. The three regions in time and space covered by the three instrumental conditions used in QENS here are labeled as ToF1, ToF2 and ToF3.

The relevant quantity we are interested in is the long-range diffusion of the center-of-mass (CoM) of the whole molecule. But, as QENS probes the dynamics on a rather local scale (few Å), the contribution of the molecule side-chains (Fig. 1) is not negligible. At a larger scale, the liquid self-organization constitutes a spatial heterogeneity that also influences the averaged IL dynamics^5^. We develop a model (SI section 1) able to describe all those dynamical modes *i.e*. fast reorientational motions of the side-chains (labeled *sc*), long-range diffusion of the CoM (*lr*) and tumbling of the whole molecule around itself (SI section 2). As this is a specificity of this system, we also pay a special attention to the transient localized motion (*loc*) due to the IL self-organization^7^. The total dynamical structure factor (SI section 1.2) of the BMIM cation is:

1 $$S(Q,\omega )\approx {I}_{1}(Q){L}_{lr}(Q,\omega )+{I}_{2}(Q){L}_{loc}(Q,\omega )+{I}_{3}(Q){L}_{sc}(Q,\omega )$$

where the *I*_*i*_(*Q*) are combinations of the form-factors describing the geometry of the motion associated to each of the dynamical modes and a parameter *p* is weighting the proportion of the side-chain protons for each of the neat or selectively deuterated sample (Fig. 1a).

In conclusion, the dynamical structure factor describing the model is composed of three Lorentzian relaxations. Each of the HWHM (Half Width at Half Maximum) are related to a unique dynamical mode. In the time domain, as directly accessed by NSE, Eq. (1) can be rewritten as:

2 $$I(Q,t)\approx {I}_{1}(Q){e}^{-t/{\tau }_{lr}(Q)}+{I}_{2}(Q){e}^{-t/{\tau }_{loc}(Q)}+{I}_{3}(Q){e}^{-t/{\tau }_{sc}(Q)}$$

where *τ*_*i*_ = *ħ*/Γ_*i*_ with *i* = loc, sc and *τ*_*lr*_ = (*D*_*lr*_ *Q* ^2^)^−1^. *D*_*lr*_ is the long-range translational diffusion coefficient inferred by the neutron methods at the molecular scale (ps-ns / 0.1–10 nm).

### Molecular scale dynamics: cation specific deuteration to challenge the robustness of the neutron model

**Figure 2:**
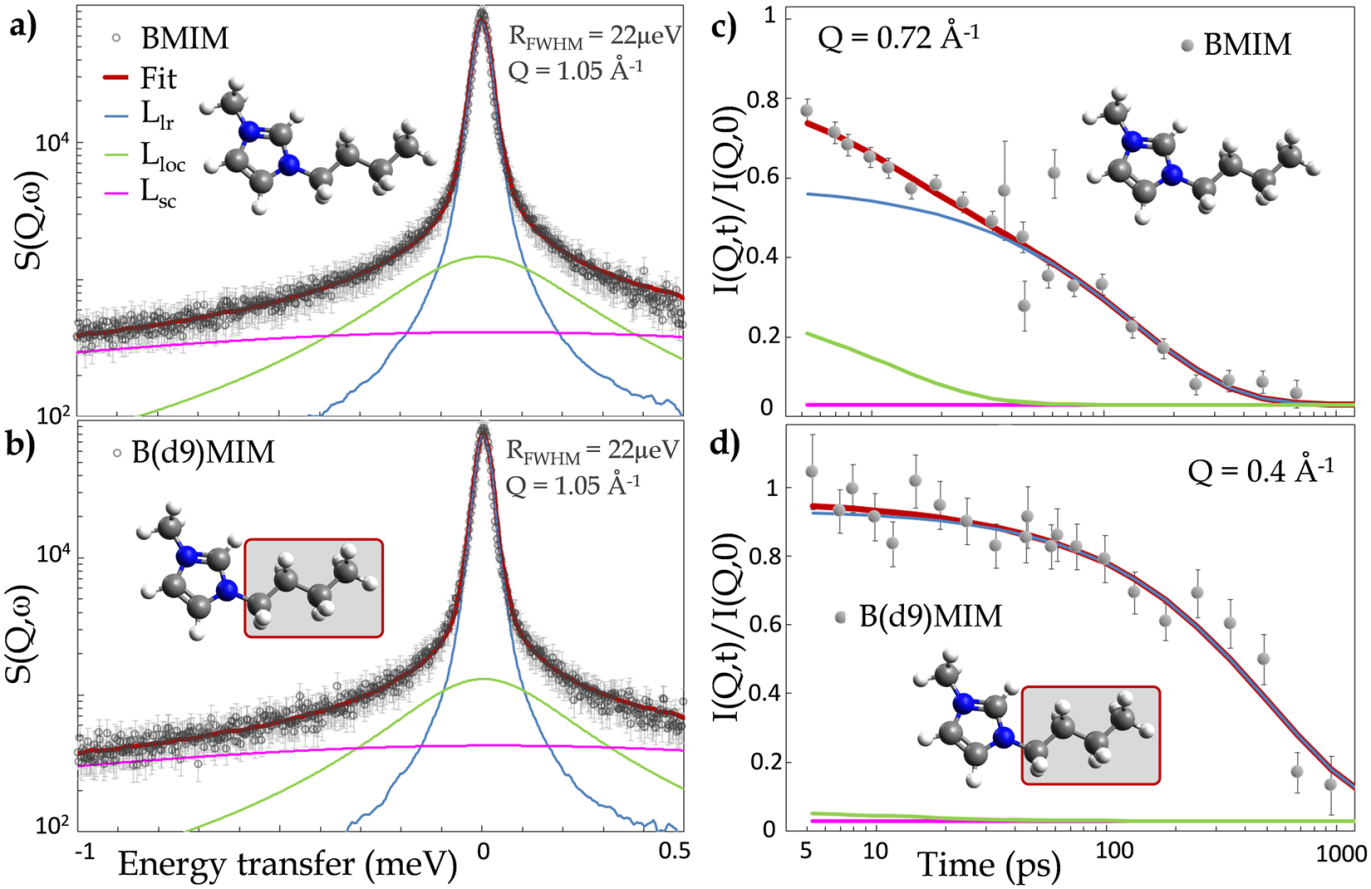
**(a)** ToF spectra (LET, ISIS, Chilton-Didcot, UK) of bulk BMIM-TFSI. The red thick line is the fit by Eq. (1) and the three dynamical contributions are shown: side-chains (Eq. S4, pink line), local diffusion within an aggregate (Eq. S5, green line) and long-range diffusion of the whole cation (Eq. S8, blue line). **(b)** Same as **(a)** but on the sample with selectively deuterated alkyl side-chain: B(d9)MIM-TFSI. **(c)** and **(d)** Same sample as **(a)** and **(b)** respectively but in the nanosecond time range by NSE (IN11, ILL, Grenoble).

Figure 2 and Fig. S2 show that the model Eq. (1) fairly well describes the QENS experimental data of the BMIM cations at 298 K. The local dynamics of the side-chains strongly contributes to the neutron spectra. In order to check that this major contribution is well accounted-for by the model, experiments have been performed on a BMIM-TFSI sample with deuterated alkyl side-chains (Fig. 1). While the statistical weight of the alkyl side-chain protons in equation Eq. S4 is *p* = 0.8 for the fully hydrogenated form of BMIM, it reduces to *p* = 0.5 for the selectively deuterated analog. The fits are in good agreement with the QENS (Figs S2, S3) and NSE (Figs S4, S5) data, for both the hydrogenated and deuterated forms of the BMIM cation. The model is therefore valid for both picoseconds and nanoseconds time ranges. The very good description of three energy resolutions ToF QENS independent data sets of the hydrogenated and deuterated BMIM cations over a broad energy and Q ranges shows the robustness of the model.

**Figure 3:**
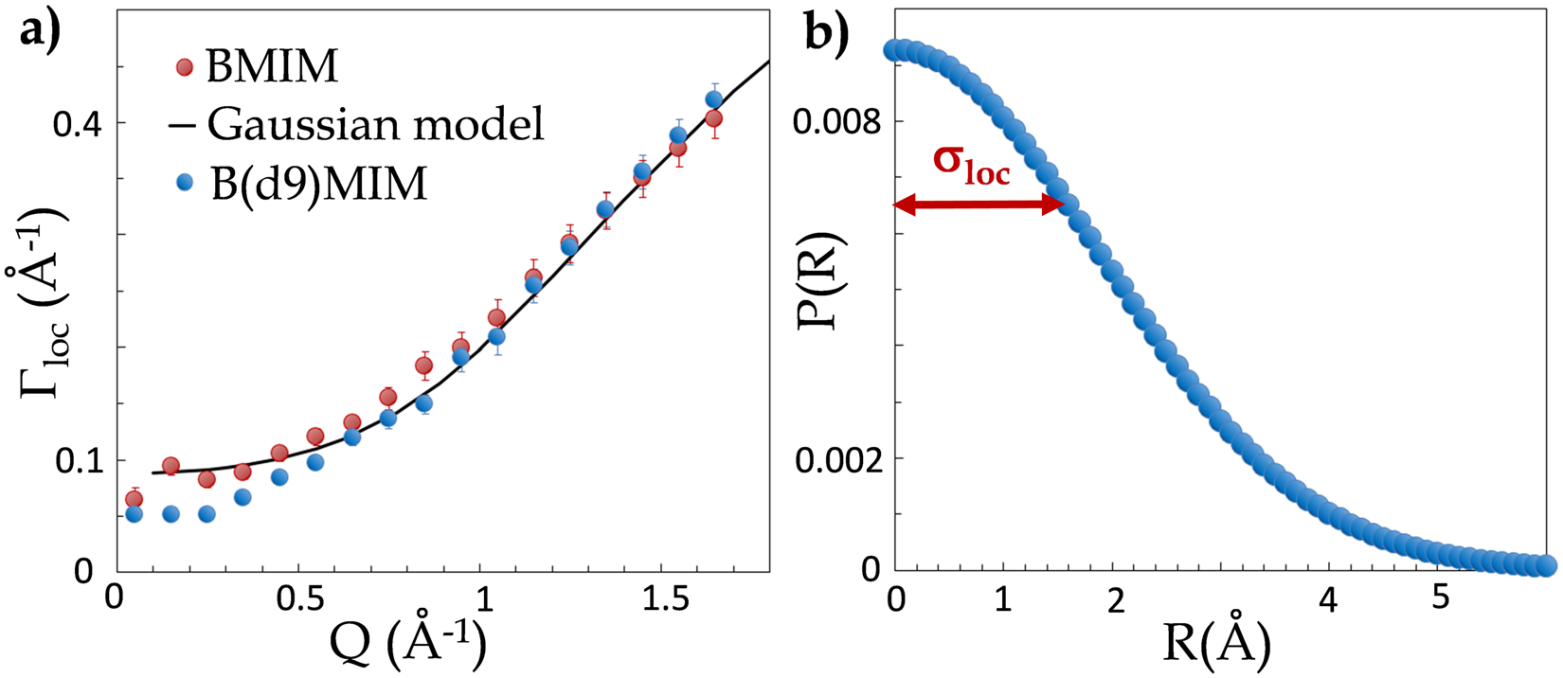
(**a**) HWHM,Γ_*loc*_, related to the diffusion of BMIM-TFSI and B(d9)MIM-TFSI within the transient nanometric IL aggregates (Eq. S6). The full line is the fit of the experimental points with the Gaussian model (see section 3 of the SI text). The local diffusion coefficient of the cation within the BMIM-TFSI nanodomains, *D*_*loc*_ = 4.8 ± 0.3 10^−5^ cm^2^/s. **(b)** Characteristic size of an aggregate: 6 × *σ*_*loc*_ = 11.4 ± 1.2 Å. (EISF eq. S7).

The Q dependence of Γ_*loc*_, the energy associated to the mode describing the translational dynamics of an IL cation within an aggregate is shown in Fig. 3a. The plateau at small Q is characteristic of a diffusion within a confining volume. The physical parameters *D*_*loc*_, *σ*_*loc*_ and *τ*, associated to this localized diffusion can be deduced from a fit of the data by the Gaussian model (SI section 3). The local dynamics of a cation can then be described as a jump diffusion with a residence time of about 1 ps within an aggregate of a total size (length of the basis of a Gaussian with standard deviation *σ*_*loc*_) of 6 × *σ*_*loc*_ ≈ 11 Å (Fig. 3b). This transient aggregation process is clearly depicted by MD simulations^4^ and is induced by the segregation of the cation polar and non-polar moieties in distinct domains. If, here, one makes the assumption that such an aggregate is spherical, we can estimate the number of cation (of characteristic size *R*_*BMIM*_), see SI section 2.2) within an aggregate to be of the order of (3 × *σ*_*loc*_ /*R*_*BMIM*_)^3^ $\simeq$ 15 molecules.

### NSE and PFG-NMR: Probing the cation CoM diffusion at the ns/nm and (ms/*μ*m) scale

**Figure 4:**
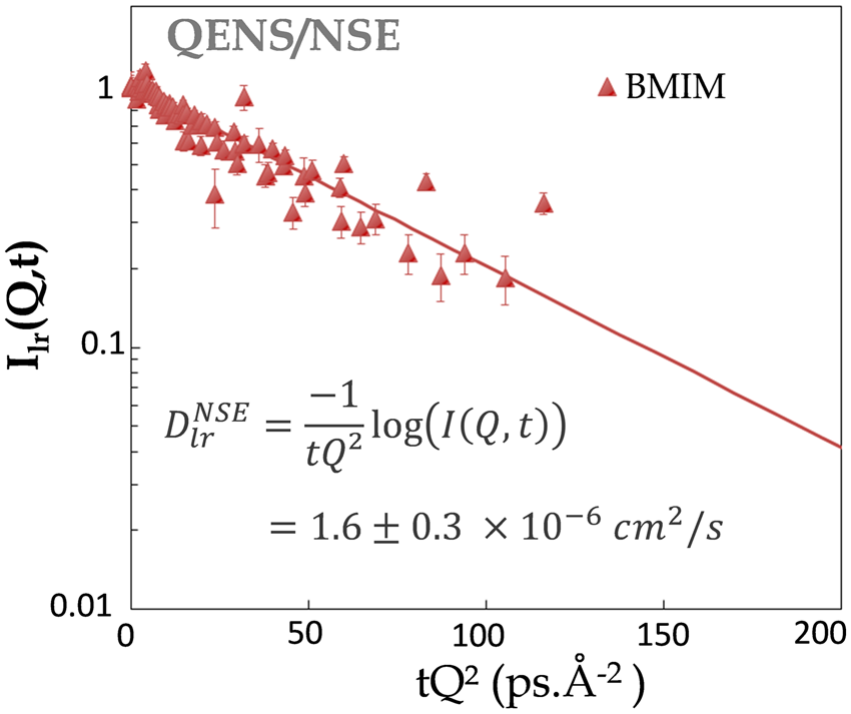
All the BMIM NSE *e* ^−*t*/*τ*^_*lr*_^(*Q*)^ long-range translational diffusion contribution term of Eq. (2) (SI section 4) merge on a single master-curve in *t*.*Q* ^2^. The slope of this curve is a direct measurement of the BMIM long-range diffusion coefficient at the nanometric scale: ${D}_{BMIM}^{NSE}=$ 1.6 ± 0.3 × 10^−6^ cm^2^/s.

To extract *D*_*lr*_, the long-range translational diffusion coefficient of the IL cation CoM, we introduce in the NSE data, the contributions of (*i*) the local diffusion within an aggregate and of (*ii*) the side-chains tumbling motion, both easily measured by ToF QENS in the ps time-scale. We show that the resulting NSE data contribution can be accounted for by a simple Q/time master curve (Fig. 4) *i.e*. that the diffusive motion associated with *D*_*lr*_ obeys a *DQ* ^2^ law. This is a strong evidence that *D*_*lr*_ is related to a genuine long-range translational motion with a translational diffusion coefficient ${D}_{lr}^{NSE}$. We also probe BMIM-TFSI on the microscopic scale (*μm* and ms) by PFG-NMR (Fig. S6). The resulting self-diffusion coefficient, ${D}_{t}^{NMR}$ = 2.7 ± 0.1 × 10^−7^ cm^2^/s, is 6 times smaller than the long-range diffusion coefficient values derived from the QENS/NSE data (SI Table 1).

### Bridging the molecular and the microscopic scales transport properties: Particle-Probe Rheology

As stated in the introduction, the ILs propensity to self-organization can make them resemble to so-called complex fluids *i.e*. materials exhibiting hierarchical structures encompassing several characteristic length-scales and experiencing thermal fluctuations. In all such physical (supercooled liquids, gels^8^…) or even biological (*crowded* cellular environments^9^) systems, the use of nanoparticle probes^10^, early suggested by de Gennes^11^, has shown tremendous success. In this paper, we introduce the use of nanoparticle probes to access the viscoelastic properties of ILs. Latex particles (radius *R*_*L*_ = 220 ± 5 nm) are highly diluted within a BMIM-TFSI sample and their translational dynamics is then studied by DLS. If a spherical particle of radius *r*_*s*_ is immersed in a fluid composed of spherical particles of radius *r*_*f*_, the long range diffusivity, *D*_*t*_, of this particle obeys the hydrodynamic relationship:

3 $${D}_{t}=\frac{{k}_{B}T}{{c}_{s}\pi \eta {r}_{s}}$$

where *k*_*B*_ is the Boltzmann constant, T the temperature, *c*_*s*_ is a constant and *η* the viscosity of the fluid. When the size of the diffusing particle is significantly larger than those of the medium (*r*_*s*_/*r*_*f*_ ≫ 1, this is the *sticking boundary* limit), *c*_*s*_ = 6 and one recovers the famous Stokes-Einstein relation. If the size of the particles are similar (*r*_*s*_/*r*_*f*_  ≈ 1 *i.e*. the *slipping boundary* limit), *c*_*s*_ lies in the range from 3 to 4^12, 13^.

**Figure 5:**
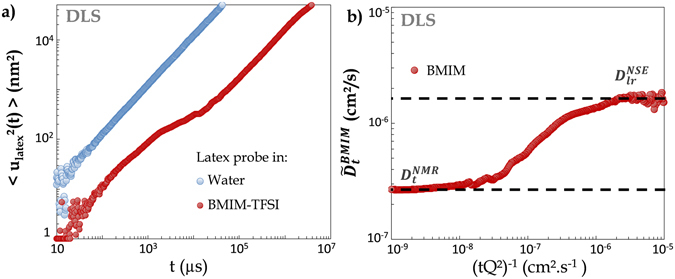
(**a**) Dynamic Light Scattering (*λ*_*DLS*_ = 647 nm, *Q* = 4.0 × 10^−3^ nm^−1^): MSD of a nanometric probe (latex, 220 nm radius) embedded in water (blue) and bulk BMIM-TFSI (red). While in water the particle MSD is proportional to time (pure Fickian diffusion process), in the IL, around 10^4^ *μs*, the probe experiences a transient cage-like localization. Then for times larger than 10^5^ *μs* it recovers a Fickian behavior. This evidences the transient nanometric structuration of the liquid. **(b)** We derive, ${\tilde{D}}_{t}^{BMIM}$ (Eq. (5)), the molecular self-diffusion coefficient of the BMIM cation. This DLS microrheology experiment is able to bridge the molecular NSE self-diffusion coefficient at short times and local scale (${D}_{lr}^{NSE}$) to the same quantity as measured at the microscopic scale: ${D}_{t}^{NMR}$.

Figure 5a shows the time dependence of the Mean-Square Displacement (MSD) $< {r}_{L/W}^{2}(t) >$ and $< {r}_{L/IL}^{2}(t) >$ of the Latex particle respectively immersed in water and in BMIM-TFSI. In water, the Latex nanoparticle MSD is proportional to time ($< {r}_{L/W}^{2}(t) >$ = $6.{D}_{t}^{L/W}{\rm{.}}t$) over the total investigated time range. This is expected for a Fickian process with a translational diffusion coefficient ${D}_{t}^{L/W}$. At short times, the MSD of Latex in the IL, $< {r}_{L/IL}^{2}(t) >$, shows a similar Fickian behavior, but at a time centered at *t* ≈ 10^4^ *μs*, the MSD plateaus for about 10 ms. This evidences a transient localization of the Latex particle in a cage of characteristic size $< {r}_{L/IL}^{2}(t) >$ = 240 *nm* ^2^. Then for times larger than *t* = 10^5^ *μs* it recovers a Fickian diffusion. Such a behavior has already been reported^14^ in a diffusing wave spectroscopy study of the motion of probe particles embedded in a semi-dilute micelles solution: at very short times the probe is governed by the local viscosity of the solvent, and at longer times, by the stress relaxation mechanisms of the micelles. In a way similar to Bellour *et al*.^14^, we interpret our data in terms of caged dynamics of the probe sphere: we see the MSD plateau as due to the dominantly elastic response of the medium followed at longer time by a cage relaxation mechanism. This similarity in the behavior of the probe in BMIM-TFSI and in a micellar system is interesting as a micelle-like structure has been proposed^15^ for the structure of an other imidazolium IL.

Another important information can also be derived from these MSD data: the intercept of the linear dependencies of $< {r}_{L/IL}^{2}(t) >$ before and after the localization event at *t* ≈ 10^4^ *μ*s shows that the Latex particles diffuse with two distinct diffusion coefficients ${D}_{st}^{L/IL}$ and ${D}_{lt}^{L/IL}$ at short (*st*) and long (*lt*) times. From Eq. (3), it is then also clear that we have evidenced a time/space scale dependent viscosity of the IL.

From the ratio of the time-dependence of the MSD shown in Fig. 5a, and using Eq. (3) one writes:

4 $$\frac{ < {r}_{L/IL}^{2}(t) > }{ < {r}_{L/W}^{2}(t) > }=\frac{{D}_{t}^{L/IL}}{{D}_{t}^{L/W}}=\frac{{\eta }_{W}}{{\eta }_{IL}}$$

where *η*_*W*_ and *η*_*IL*_ are respectively the viscosity of water and of the IL. So far, as we have considered the behavior of the Latex particles, we have used Eq. (3) in the *sticking boundary* limit. By combining Eq. (4) with Eq. (3) but now in its *slipping boundary* limit, we directly assess ${\tilde{D}}_{t}^{BMIM}$, the translational diffusion coefficient of the BMIM cation at the molecular scale:

5 $${\tilde{D}}_{t}^{BMIM}=\frac{{k}_{B}T}{{c}_{BMIM}{R}_{BMIM}}\mathrm{.}\frac{1}{\pi {\eta }_{W}}\mathrm{.}\frac{ < {r}_{L/IL}^{2}(t) > }{ < {r}_{L/W}^{2}(t) > }$$

where *c*_*BMIM*_ is a constant and *R*_*BMIM*_ is the radius of the BMIM cation as considered as a sphere. The viscosity of water^16^ at 25 °C is *η*_*W*_ = 0.913 cP. The group of Watanabe has performed a thorough study of the physico-chemical properties of BMIM-TFSI^17^. They provide an estimate of the two key parameters of Eq. (5): *c*_*BMIM*_ = 3.4 and *R*_*BMIM*_ = 3.3 Å. This later quantity is estimated from the radius of an equivalent sphere matching the 3D structure of the cation as deduced from *ab-initio* calculations. For the sake of consistency of our QENS/NSE, NMR and DLS data, we use *R*_*BMIM*_ = 2.3 Å (see SI section 2.2). This 30% difference with the value proposed by Tokuda *et al*.^17^ seems acceptable as the structure of the BMIM cation (Fig. 1a) is far from spherical. From Eq. (5) the DLS derived MSD experimental values of Fig. 5a can be turned into the quantity we focus on: the long-range translational diffusion coefficient of the BMIM molecule. We write this DLS derived value ${\tilde{D}}_{t}^{BMIM}$. As shown in Fig. 5b, ${\tilde{D}}_{t}^{BMIM}$ perfectly bridges the one order of magnitude difference of diffusion coefficient of the BMIM molecule at the molecular scale (as inferred from QENS/NSE) to the one measured at the microscopic scale by PFG-NMR. To the deep of our knowledge, this multi-scale analysis provides the first experimental evidence of the transient localization of the BMIM cation molecule.

## Discussion

Due to their unique physico-chemical properties, Ionic Liquids have been proposed for fundamental and industrial applications in fields as different as catalysis, liquid/liquid extraction, processing of bio to nuclear materials, to cite a few examples^2^. When addressing the properties of ILs, two key parameters have to be considered: the quantity of gas^18, 19^ and/or water^20^ solubilized in the IL coming from air and atmospheric moisture. BMIM-TFSI is known to show *CO*_2_ and *O*_2_ absorption capabilities^21^. Also, the viscosity of BMIM-TFSI has been shown to be strongly dependent on the water content^20^. In order to minimize any contamination by these impurities, we have paid special attention to the BMIM-TFSI handling (see *Materials and Methods*).

We note in particular that, for the particle-probe rheology experiment, the water content introduced in the BMIM-TFSI sample by the latex solution dispersion induces a viscosity change of less than 1%: 0.065 *vs* 0.064 Pa.s^20^ for respectively the pure BMIM-TFSI and the BMIM-TFSI sample with a water content of 0.2 × 10^−3^ g/g at 293 K (see *Materials and Methods*). To conclude on the absence of impurities in the BMIM-TFSI sample, we also note that the cation self-diffusion coefficient we measure by PFG-NMR (${D}_{t}^{NMR}$ = 2.7 ± 0.1 × 10^−7^ cm^2^/s) is perfectly consistent with the one published earlier by Tokuda *et al*.^17^: 2.74 × 10^−7^ cm^2^/s at 298 K.

**Figure 6:**
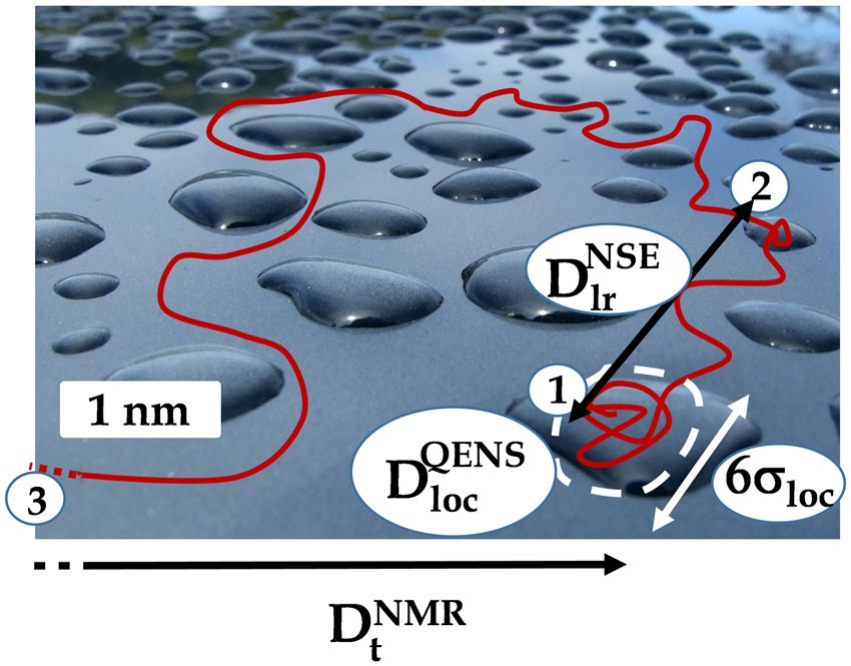
Two dimensional cross-section artist view (image courtesy of Adrian van Leen, rgbstock.com) of the nanostructuration of an IL and the consequences on the transport properties (see text). Aggregated micelle-like and non-aggregated regions of the liquid are respectively represented as dark and light blue regions. The path of a cation molecule within the system is indicated by a red line. Starting from the confined motion within an aggregate (1), a BMIM cation experiences a long-range diffusion in a non-aggregated part of the system (1) to (2) but at larger scale it labyrinths around the aggregates (1) to (3), resulting in an apparent small diffusion coefficient. The path within an aggregate (1), from (1) to (2), and from (1) to (3) are respectively probed by QENS, NSE, and NMR. The dashed line indicates that the corresponding NMR path extends on a much larger scale than the zoomed region shown here. This picture is a snapshot view of a process that in reality is a dynamical equilibrium where a cation can successively be part of an aggregated or not aggregated region.

We experimentally directly show the existence of two distinct viscosities in BMIM-TFSI. Altogether, based on the spatial and dynamical multi-scale analysis presented here, we conclude the following behavior of the IL. At the nm/ns scale, a cation diffuses within BMIM-TFSI nanodomains of characteristic size 6 × *σ*_*loc*_ = 11.4 ± 1.2 Å with a diffusion coefficient *D*_*loc*_ = 4.8 ± 0.3 10^−5^ cm^2^/s. Then after the *dissolution* of the transient aggregates, at the tens of nm and ns probed by NSE, the cation experiences a pure long-range translational diffusion with a diffusion coefficient ${D}_{lr}^{NSE}$ = 1.6 ± 0.3 × 10^−6^ cm^2^/s. At the *μm* and ms scale probed by PFG-NMR, the tortuous pathway in between the aggregates, results in an apparent one order of magnitude (${D}_{lr}^{NSE}$/${D}_{t}^{NMR}$ = 6 ± 1) smaller diffusion coefficient ${D}_{t}^{NMR}$ = 2.7 ± 0.1 × 10^−7^ cm^2^/s. A two dimensional view of the IL segregation and an illustration of this multi-scale transport process are shown on Fig. 6. We need to note that, following a *k*_*B*_ *T* driven density fluctuation leading to the formation of an aggregate, a cation can be trapped within this aggregate for a transient period of time.

Here, we focus only on the time dependence of the phenomenon, but a promising extension of the method could be the observation of the MSD probe size-dependence (still detected by DLS). The time and MSD *loci* of the plateau on a MSD vs time plot, as shown in Fig. 5a, should be strongly influenced by the particle size (*R*_*P*_), and we expect that all the plateau positions should fall on a MSD *vs* *Q* *R*_*P*_ master curve. Such a Guinier-like representation seems a possible route to directly extract the characteristic size of the IL aggregates.

It should be noted that the particle probe strategy presented here for the characterization of the nanometric self-organization of BMIM-TFSI could be easily generalized. A possible limitation is nevertheless probably the colloidal stability of the probe within the liquid. Also, specific interaction of the ILs with the probe surface have been reported in particular in the case of silica nanoparticles^22^. In the present case of a Latex probe such limitation of the method can be nevertheless dismissed as the diffusion coefficient as measured with and without Latex probes, by DLS and (NSE/NMR) respectively, are in full agreement.

In electrolytes, the ionic conductivity and the transport properties are directly related by the Nernst-Einstein relation. Hence, since they represent transient energy barriers for the long-range diffusion of the charges, the ILs nanostructuration and the related viscosity fluctuations, that we evidence here, have important detrimental consequences on the ionic conductivity. A direct way to circumvent the spontaneous formation of aggregates within the ILs would be to confine the liquid in a porous material with pores smaller than the characteristic size of the aggregates. Following experimental data^23^, recent MD results indicate^24^ that imposing confinement of ILs in Carbon NanoTubes (CNT) with diameter under 2 nm exalts the transport properties by several orders of magnitude. To date, no evidence shows that such gigantic transport properties are solely due to the frustration of the spontaneous nano-aggregates formation. These study nevertheless provide an interesting perspective to take advantage of macroscopically oriented one dimensional (1D) nanometric materials to enhance the ionic conductivity of ILs and make such systems very efficient batteries separators^25^.

## Conclusion

We have evidenced that the transport properties of ILs are scale dependent, showing a one order of magnitude difference of the translational diffusion coefficient, depending whether it is inferred at the molecular (ps-ns/Å) or at the microscopic (ms/*μ*m) scales. We assign this apparent discrepancy to a specific property of ILs: spontaneous transient nanometric self-assembly. We have used Particle Probe Micro-Rheology to experimentally catch the ILs nano-segregation phenomenon and qualitatively and quantitatively bridge the transport properties measured at the local scale (here by QENS and NSE) to the same quantity assessed at the microscopic scale by PFG-NMR. This method has general relevance as a way to follow both in space and time processes at play in self-assembling systems.

## Materials and Methods

All the experimental data are measured at 298 K.

### BMIM-TFSI

1-butyl-3-methyl imidazolium bis(trifluoromethanesulfonyl imide (BMIM-TFSI, electrochemical grade, Purity ≥ 99.9%, anhydrous, *H*_2_ *O* ≤ 0.005%) was purchased from Solvionic. To avoid gas solubility the BMIM-TFSI was transferred from the initial container to a number of 5 ml vials then crimped with a septum. This operation was done in a glove box. The BMIM-TFSI samples used for all the experiments described in the present paper were extracted from these vials using with needles and syringes.

### Neutron scattering

QENS measurements were performed on the LET cold chopper spectrometer (ISIS, Chilton-Didcot, UK)^26^ in its repetition rate multiplication mode^27^. With this setting, successive wavelength bands are selected within each of the incident neutron pulses. For the results shown in this paper, the incident wavelength bands were *λ*_0_ = 5.2, 8.0 and 10.8 Å respectively resulting in 81, 22, 13 *μ*eV energy resolutions and [0.06–2.28], [0.04–1.05] and [0.03–0.75] Å^−1^ Q ranges. To extend the timescale probed with LET up to 1.5 ns (*i.e*. 1 *μ*eV resolution), we performed an NSE experiment on the spectrometer IN11 (ILL, Grenoble, France) with an incident wavelength of 5.5 Å. Two positions of the multi-detector have been used (20° and 50°) to cover a total Q range between 0.1 and 1.25 Å^−1^. Samples were enclosed in Aluminum cylindrical containers sealed with indium. A flat piece of Vanadium has been used to measure the resolution function and correct the S(Q,*ω*) spectra for the detectors efficiency.

### Particle-probe rheology

The experimental set-up has been described in a preceding paper^28^. In the present study, the polystyrene latex particle (radius 220 ± 5 nm) weight fraction of the initial batch was 5.6 10^−2^ g/g as determined by weighing after evaporation of water. A volume of 0.5 *μl* of the mother latex solution was incorporated in 1.5 cm^3^ of BMIM-TFSI and diluted in several steps down to a latex concentration of 0.2 10^−3^ g/g. The same procedure was used to prepare the latex solution in water. The light scattering measurements were performed with a home made spectrometer using a laser wavelength of *λ*_*DLS*_ = 647 nm.

### PFG-NMR

The measurements have been performed using a spin echo sequence, and a ^1^H probe. The BMIM cation self-diffusion coefficient, *D*_*S*_, was determined according to the following equation:

$$A(g)/A\mathrm{(0)}={\rm{\exp }}(-{\gamma }^{2}{g}^{2}{\delta }^{2}{D}_{S}({\rm{\Delta }}-\delta /\mathrm{3))}$$

where A, *γ*, g, *δ* and Δ stand for the signal areas, the gyromagnetic ratio of the investigated nucleus, the intensity of the magnetic field gradient, the duration of the gradient pulse and the diffusion time respectively. The magnitude of the pulsed field gradient was varied as 0 ≤ *g* ≤ 1000 G/cm; *δ* was set between 1 ms and 3 ms; Δ was set between 10 ms and 50 ms.

## Electronic supplementary material

ESI file
