# Supplementary material for: Ionic Liquids: evidence of the viscosity scale-dependence
Source: Sci Rep. 2017 May 22;7:2241. doi: 10.1038/s41598-017-02396-7 (PMC5440414; doi:10.1038/s41598-017-02396-7)
Supplement: Supplementary file 1 — ESI file [file 41598_2017_2396_MOESM1_ESM.pdf]

Supplementary Information

Ionic Liquids: evidence of the viscosity scale-dependence.

Q. Berrod, F. Ferdeghini, J.-M. Zanotti, P. Judeinstein, D. Lairez, V. García Sakai, O. Czakkel, P. Fouquet and D. Constantin

1 Modeling the Molecular scale dynamics (ps-ns/Å-nm)

Due to the large incoherent neutron scattering cross-section of the hydrogen element, protonated samples scatter mainly incoherently. In this case, the QENS experimental intensity is directly related to the incoherent dynamical structure factor $S_{inc}(Q,\omega)$, the Fourier transform over space and time of the self-correlation function $G_s(r,t)$ of the nuclei in the system[1]:

$$S_{inc}(Q,\omega) = \int G_s(\vec{r},t) e^{i(\vec{Q}\vec{r}-\omega t)} d\vec{r} dt \tag{S1}$$

If these particles do not experience perfect ergodic dynamics, $G_s(r,t=\infty)$ is non-zero, so that $S_{inc}(Q,\omega)$ shows an elastic component:

$$S_{inc}(Q,\omega) = A(Q)\,\delta(\omega) + (1-A(Q))\,L(Q,\omega) \tag{S2}$$

where $A(Q)$, the Elastic Incoherent Structure Factor (EISF), is the Fourier transform of the $G_s(r,t=\infty)$ loci. In the case of a particle diffusing in a reduced volume of space, such as a sphere or a cylinder, $A(Q)$ is simply the form factor of this confining volume. $L(Q,\omega)$ is related to the time dependence of the particle auto-correlation function. This term is generally described by a sum of Lorentzian lines[1] and is in practice usually well accounted for, by a single Lorentzian line with a Half-Width-at-Half-Maximum (HWHM), $\Gamma(Q,\omega)$.

1.1 A multi-components model

1.1.1 Modeling the side-chains dynamics

In the reference frame of the IL cation, along with free and/or jump rotation of the methyl group carried by the imidazolium ring, the side-chain methylene group (Fig.1) experiences rapid (ps) and local (1-2 Å) dihedral reorientations known to significantly contribute to the QENS signal. Within the statistics of a QENS experiment, it is not possible to finely describe the details of such a complex contribution. In order to limit the number of parameters in our model, we have therefore made the choice to account for the side-chains dynamical contribution by a single *average* dynamical structure factor:

$$S(Q,\omega)_{inc}^{sc} = A_{sc}(Q)\,\delta(\omega) + (1-A_{sc}(Q))\,L_{sc}(Q,\omega) \tag{S3}$$

where $A_{sc}(Q)$ is the EISF of the relaxation, while $L_{sc}(Q,\omega)$ is a Lorentzian function with Γ_{sc} HWHM (the sc subscript stands for *side-chain*). As discussed later-on in this paper, we have taken advantage of specific deuteration of the side-chain to check the validity of this approximation.

Next to the side-chain protons, the imidazolium protons experience no dynamics. In its own reference frame, $S(Q,\omega)_{inc}^{CF}$ (CF stands for *Cation Frame*), the total dynamical structure factor of an IL cation is:

$$S(Q,\omega)_{inc}^{CF} = (1-p)\delta(\omega) + pS(Q,\omega)_{inc}^{sc} \tag{S4}$$

where p is the fraction of the sole side-chains protons. In the case of fully hydrogenated BMIM $p = 12/15 = 0.8$. As the neutron incoherent cross-section of deuterium is negligible compared to the one of hydrogen ($\sigma(D) = 2$ barns $<< \sigma(H) = 80$ barns), in the case of a BMIM cation with a fully deuterated alkyl side-chain, $p = 3/6 = 0.5$.

1.1.2 Diffusion within aggregates

Burankova *et al.*[2] have proposed that a confinement space could account for a localization of the molecules within the IL nanometric aggregates. This development is based on the so-called Gaussian model developed by Volino *et al.*[3], which describes the translational motion of particles confined in spaces with soft boundaries. At small Q, the HWHM of this line tends to a plateau $\Gamma_{loc}(Q \to 0) = \hbar D_{loc}/\sigma_{loc}^2$, while for large Q, it recovers the jump diffusion law until a saturation related to τ, the residence time between two successive jumps: $\Gamma_{loc}(Q \to \infty) = \hbar/\tau$.

To reduce the complexity, our model use a single Lorentzian function to describe the diffusion within aggregates (with a single fitting parameter: Γ_{loc}). The corresponding dynamical structure factor is:

$$S(Q,\omega)_{inc}^{loc} = A_{loc}(Q)\,\delta(\omega) + (1 - A_{loc}(Q))\,L_{loc}(Q,\omega) \tag{S5}$$

where $L_{loc}(Q,\omega)$ is a Lorentzian function:

$$L_{loc}(Q,\omega) = \frac{1}{\pi}\frac{\Gamma_{loc}(Q)}{\Gamma_{loc}^2(Q) + \omega^2} \tag{S6}$$

and $A_{loc}(Q)$ is the EISF[3]:

$$A_{loc}(Q) = e^{-Q^2\sigma_{loc}^2} \tag{S7}$$

Γ_{loc} and $A_{loc}(Q)$ are then fitted with the Gaussian dynamical structure factor and the Gaussian EISF, respectively (Fig.3, see SI.3 for details). The total isotropic distance visited by the confined particle is of the order of $6.\sigma_{loc}$ ($\pm$ 3 standard deviations of the Gaussian EISF function).

1.1.3 Cation center-of-mass long-range diffusion

The dynamical structure factor of this mode is simply the Fick's law in the reciprocal space[1]:

$$S(Q,\omega)_{inc}^{lr} = \frac{1}{\pi}\frac{\Gamma_{lr}(Q)}{\Gamma_{lr}^2(Q) + \omega^2} \tag{S8}$$

where Γ_{lr}, the HWHM of this Lorentzian, is directly proportional to D_{lr}, the long-range self-diffusion coefficient:

$$\Gamma_{lr}(Q) = D_{lr}Q^2 \tag{S9}$$

The Equation S9 is the so-called DQ^2 *law*.

1.2 Derivation of the total dynamical structure factor

Three contributions describe the dynamics of an IL cation: side-chain motions, local diffusion within aggregates and long-range diffusion. As they occur in different time windows, we suppose that they are independent. $S(Q,\omega)_{inc}^{T}$, the total dynamical structure factor, is therefore a convolution of the dynamical structure factor Eq.S4, S5 and S8 related to these individual modes:

$$\begin{aligned} S(Q,\omega)_{inc}^{T} &= S(Q,\omega)_{inc}^{CF} \otimes S(Q,\omega)_{inc}^{loc} \otimes S(Q,\omega)_{inc}^{lr} && \text{(S10)} \\ &= I_A(Q)\,L_{lr}(Q,\omega) + I_B(Q)\,L_{lr+Loc}(Q,\omega) \\ &\quad + I_C(Q)\,L_{lr+sc}(Q,\omega) \\ &\quad + I_D(Q)\,L_{lr+Loc+sc}(Q,\omega) && \text{(S11)} \end{aligned}$$

where $L_{x+y}(Q,\omega)$ is a Lorentzian line of HWHM $\Gamma_{x+y} = \Gamma_x + \Gamma_y$ and

$$\begin{aligned} I_A(Q) &= pA_{loc}(Q)A_{sc}(Q) + (1-p)A_{loc}(Q) && \text{(S12)} \\ I_B(Q) &= pA_{sc}(Q)(1 - A_{loc}(Q)) \\ &\quad + (1-p)(1 - A_{loc}(Q)) && \text{(S13)} \\ I_C(Q) &= pA_{loc}(Q)(1 - A_{sc}(Q)) && \text{(S14)} \\ I_D(Q) &= p(1 - A_{sc}(Q))(1 - A_{loc}(Q)) && \text{(S15)} \end{aligned}$$

The three dynamical contributions of equation S11 take place on different time ranges. The side-chain reorientational and dihedral motions are faster than the local diffusion witch is itself expected to be faster than that the long range one so that $\Gamma_{sc} \gg \Gamma_{loc} \gg \Gamma_{lr}$. Equation S11 can then be simplified:

$$S(\mathbf{Q},\omega)_{inc}^{cation} \approx I_1(\mathbf{Q})\,L_{lr}(\mathbf{Q},\omega) + I_2(\mathbf{Q})\,L_{loc}(\mathbf{Q},\omega) + I_3(\mathbf{Q})\,L_{sc}(\mathbf{Q},\omega) \tag{S16}$$

with:

$$I_1(\mathbf{Q}) = pA_{loc}(\mathbf{Q})A_{sc}(\mathbf{Q}) + (1-p)A_{loc}(\mathbf{Q}) \tag{S17}$$
$$I_2(\mathbf{Q}) = pA_{sc}(\mathbf{Q})(1-A_{loc}(\mathbf{Q})) + (1-p)(1-A_{loc}(\mathbf{Q})) \tag{S18}$$
$$I_3(\mathbf{Q}) = p(1-A_{sc}(\mathbf{Q})) \tag{S19}$$

In conclusion the dynamical structure factor proposed by this model is composed by three Lorentzian relaxations whose each HWHM are linked to a unique dynamical mode while the intensities are combination of the different EISF.

In the time domain as directly accessed by NSE Eq.1 can be rewritten as:

$$I(Q,t) \approx I_1(Q)e^{-t/\tau_{lr}(Q)} + I_2(Q)e^{-t/\tau_{loc}(Q)} + I_3(Q)e^{-t/\tau_{sc}(Q)} \tag{S20}$$

where $\tau_i = \hbar/\Gamma_i$ with i=loc, sc and $\tau_{lr} = (D_{lr}Q^2)^{-1}$. D_{lr} is the long-range tranlational diffusion coefficient inferred by the neutron methods at the molecular scale (ps-ns / 0.1-10 nm).

2 Rotation of the whole cation around its center-of-mass

2.1 Theoretical aspects

In the framework of the Sears approximation[4], $I(Q,t)_s^R$, the self (incoherent) intermediate scattering function of an isotropic rotation over a sphere of radius b writes:

$$I(Q,t)_s^R = j_0^2(Qb) + \sum_{l=1}^{\infty}(2l+1)j_l^2(Qb)F_l(t) \tag{S21}$$

The time-dependent term $F_l(t)$ is the first order rotational auto-correlation function:

$$F_l(t) = \langle P_l(cos\alpha(t))\rangle \tag{S22}$$

where $\alpha(t)$ is the angle between $\vec{u}(t)$, accounting for the particle orientation at time t = 0 and its orientation at time t later. P_l is the Legendre polynomial of degree l. For times longer than few ps, $F_1(t)$ et $F_2(t)$ Eq.S22 simplifies by just considering two correlation times $3\tau_1$ et τ_1 :

$$I(Q,t)_s^R = j_0(Qb)^2 + 3j_1(Qb)^2e^{-t/3\tau_1} + 5j_2(Qb)^2e^{-t/\tau_1} + \cdots \tag{S23}$$

The correlation times related to the BMIM translational diffusive motions, within an aggregate or long-range, and the tumbling of the whole molecule are supposed to be significantly different, so that can one consider these modes as being independent. From Eq.S11, in ToF QENS, the total dynamical structure factor is then:

$$S(\mathbf{Q},\omega)_{inc}^{trans+tumbling} = S(\mathbf{Q},\omega)_{inc}^{cation} * \mathscr{F}\mathscr{T}\left[(I(Q,t)_s^R,t)\right] \tag{S24}$$

where $\mathscr{F}\mathscr{T}$ denotes the Fourier Transform over time. For NSE, from Eq.S20, in the time domain the equation above is:

$$I(Q,t)^{trans+tumbling} = I(Q,t).I(Q,t)_s^R \tag{S25}$$

2.2 Numerical application in the case of BMIM

The dynamics of bulk BMIM-TFSI has recently been investigated by Nuclear Magnetic Resonance Relaxation Dispersion (NMRD) and PFG-NMR over a wide range of temperatures[5]. At 298 K, Seyedlar et al. measure the room temperature Rotational correlation times: τ_R=400 ps.

Tokuda *et al.* propose radius of the BMIM molecule: R_{BMIM}= 3.3 Å. This later quantity is estimated from the radius of an equivalent sphere matching the 3D structure of the cation as deduced from *ab-initio* calculation. For the sake of consistency of our QENS/NSE, NMR and DLS data, we use R_{BMIM}= 2.3 Å. This 30% difference with the value proposed by Takuda *et al.* seems acceptable as the structure of the BMIM cation (Fig.1a) is far from spherical.

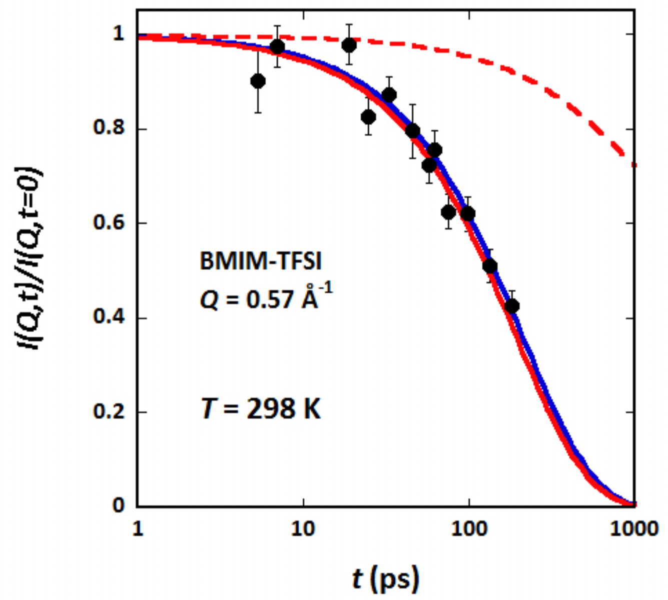

Figure S1. NSE spectrum of BMIM (the contribution of TFSI is negligible), at Q = 0.57 Å^{-1} and 298 K. The dotted red line is the contribution Eq.S23 of the isotropic rotation, with a radius $b = R_{BMIM}$ = 2.3 Å, of the BMIM cation around its center-of-mass with a correlation time[5] τ_R = 400 ps. The full blue and red lines are respectively the total intermediate scattering function of BMIM if the tumbling motion of the molecule is neglected (Eq.S20)or taken into account (Eq.S25). The situation is the same if ones considers $b = R_{BMIM}$ = 3.3 Å.

At the highest ToF QENS energy resolution (see the Methods section) used in this study, the maximum correlation accessible time is of the order of few tens of ps. A correlation time of τ_R = 400 ps is therefore detected as a extremely narrow Lorentzian line whose HWHM is negligible compared to the other Lorentzian contributions. The total dynamical structure factor of BMIM then reduces to Eq.S11.
As shown in Fig.S1, within the statistics of a NSE experiment, also in the time domain, the molecule tumbling can be neglected and the BMIM intermediate scattering function reduces to Eq.S20.

3 Implementation of the Gaussian model

To determine D_{loc}, σ_{loc} and τ, $L_{loc}(Q,\omega)$ is fitted with the Gaussian model in the time domain.
The intermediate scattering function of the Gaussian model is:

$$I(Q,t)_{inc}^{loc} = exp\left(-Q^2\sigma_{loc}^2\right)\left(1 - exp\left(\frac{-D_{loc}t}{\sigma_{loc}^2(1+2D_{loc}Q^2\tau)}\right)\right) \quad \text{(S26)}$$

and the intermediate scattering function corresponding to the diffusion within the aggregates is:

$$L(Q,t)_{inc}^{loc} = exp\left(\frac{-t}{\tau_{loc}}\right) \quad \text{(S27)}$$

where $\tau_{loc} = \hbar/\Gamma_{loc}$.
$(1 - A_{loc}(Q))L(Q,t)_{inc}^{loc}$ is then fitted with $I(Q,t)_{inc}^{loc} - A_{loc}(Q)$ to obtain the Gaussian parameters D_{loc}, σ_{loc} and τ.

4 Inferring the molecular long-range translational diffusion at the molecular scale from the NSE data

Staring from Eq.S20, the long-range tranlational diffusion coefficient at the molecular scale (ps-ns / 0.1-10 nm) is directly inferred from the NSE data corrected by the local short times contributions as measured by ToF QENS according to:

$$e^{-t/\tau_{lr}(Q)} \approx \left[I\left(Q,t\right)_{inc}^{cation} - I_2\left(Q\right)e^{-t/\tau_{loc}(Q)} - I_3\left(Q\right)e^{-t/\tau_{sc}(Q)}\right]/I_1\left(Q\right) \quad \text{(S28)}$$

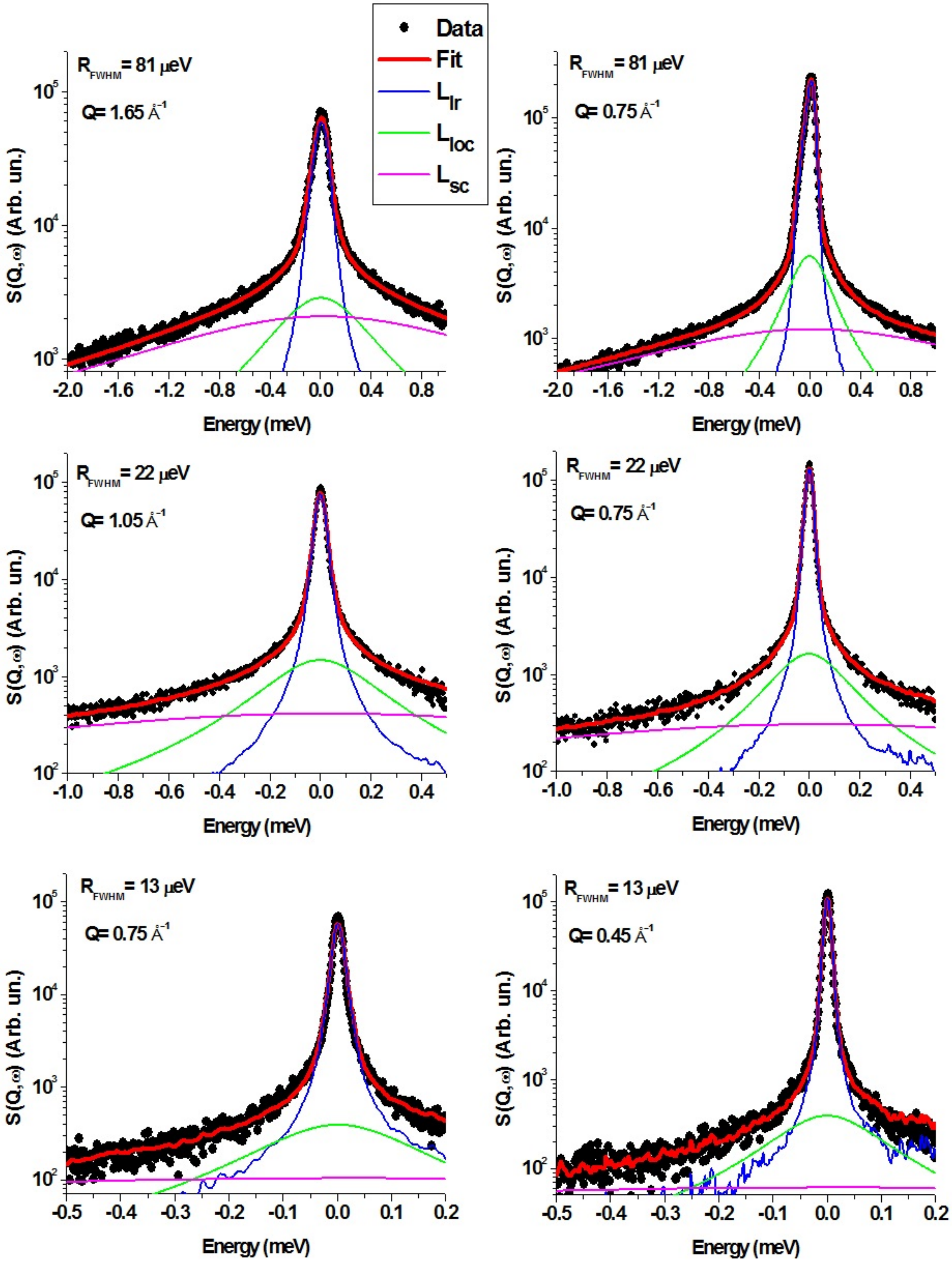

Figure S2. Selected QENS spectra (LET, ISIS, Chilton-Didcot, UK) of bulk BMIM-TFSI at 298 K as measured on LET at three energy resolutions 81, 22 and 13 μeV from top to bottom. The red thick line is the fit Eq.S20 and the three dynamical contributions are shown: side-chains (Eq.S4, pink line), local diffusion within an aggregate (Eq.S5, green line) and long-range diffusion of the whole cation (Eq.S8, blue line).

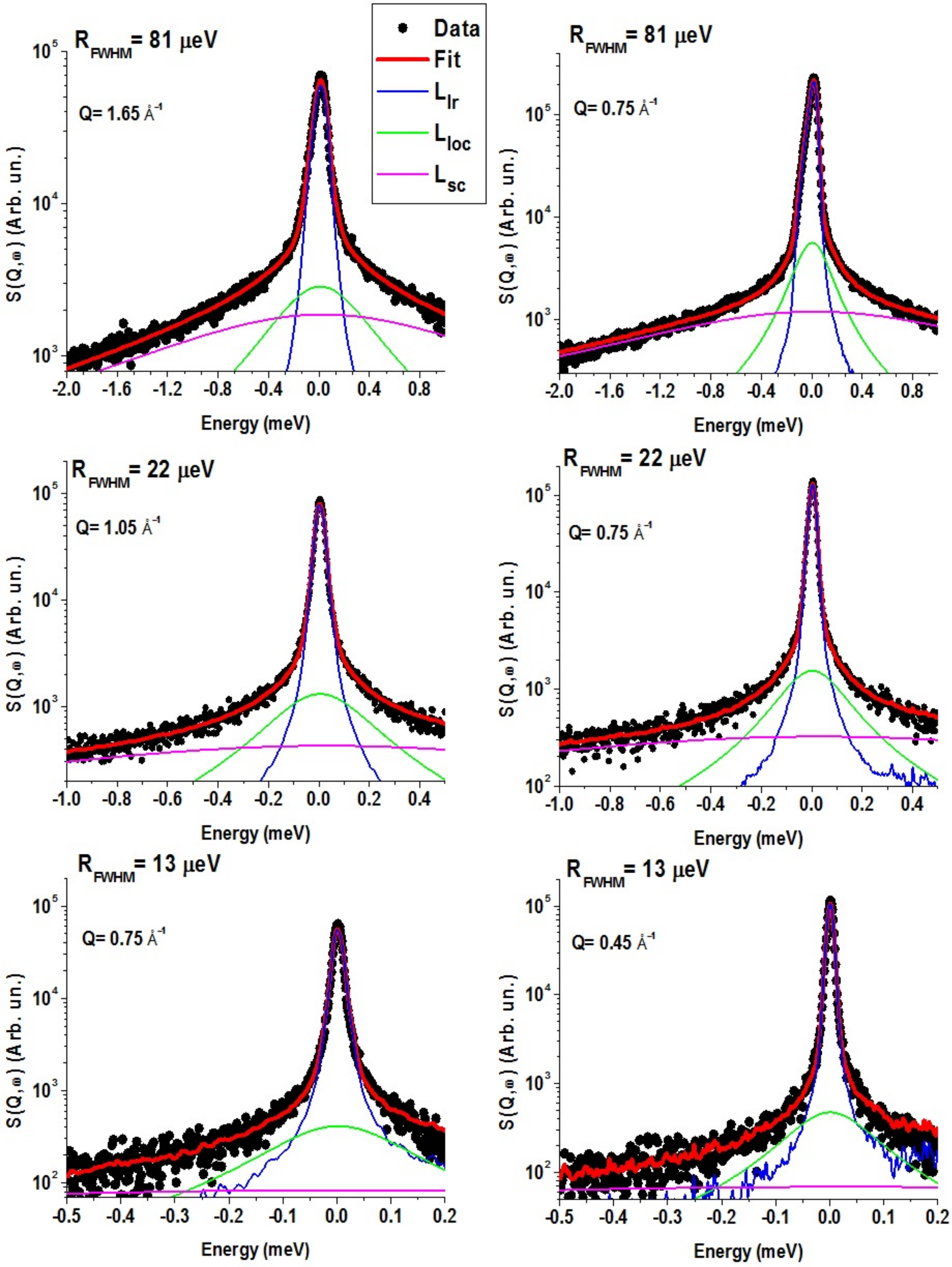

Figure S3. Selected QENS spectra (LET, ISIS, Chilton-Didcot, UK) of bulk B(d9)MIM-TFSI at 298 K as measured on LET at three energy resolutions 81, 22 and 13 μeV from top to bottom. The red thick line is the fit Eq.1 and the three dynamical contributions are shown: side-chains (Eq.S4, pink line), local diffusion within an aggregate (Eq.S5, green line) and long-range diffusion of the whole cation (Eq.S8, blue line).

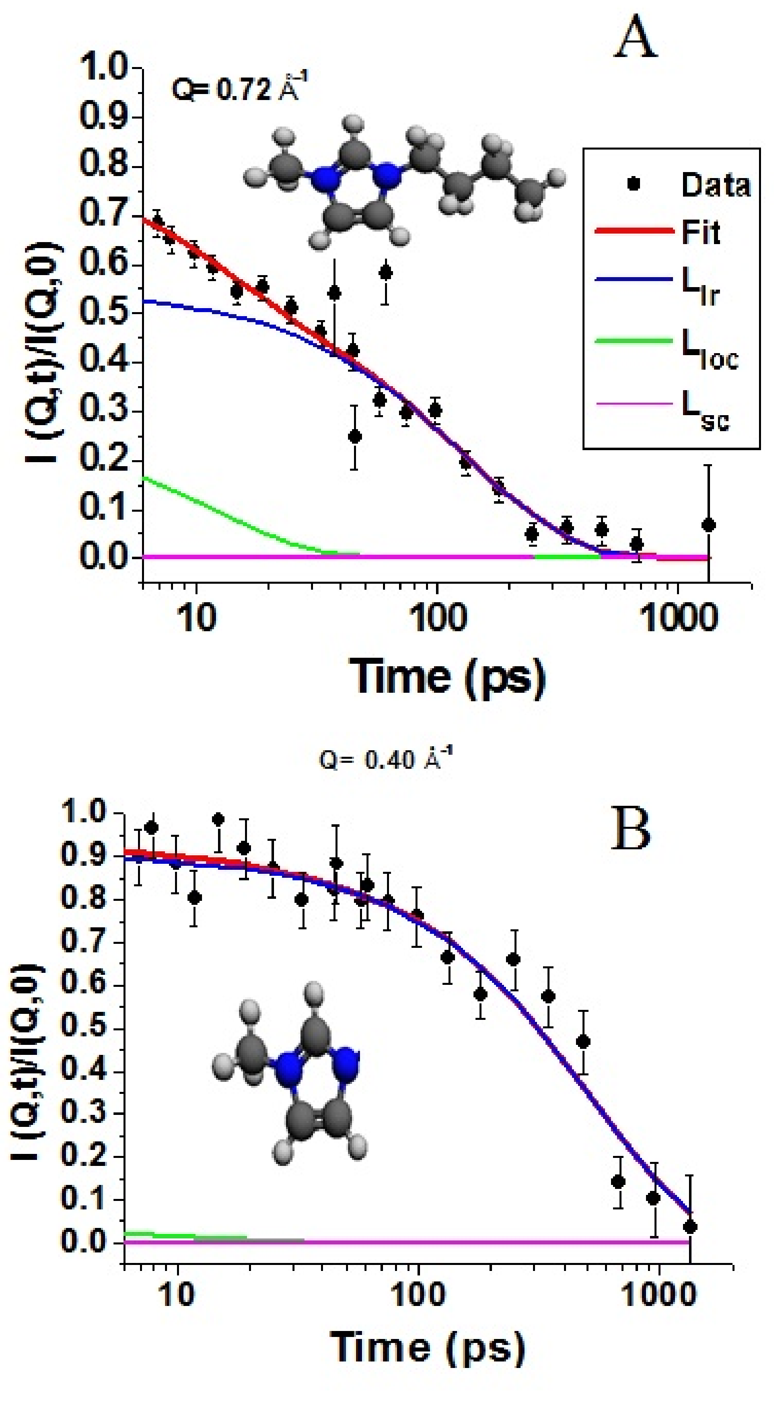

Figure S4. NSE spectra of bulk BMIM-TFSI (top) and B(d9)MIM-TFSI (bottom) at 298 K. The total fit according to Eq.2 is shown as the thick red line. The individual dynamical contributions are shown with the same color code as in Fig.2.

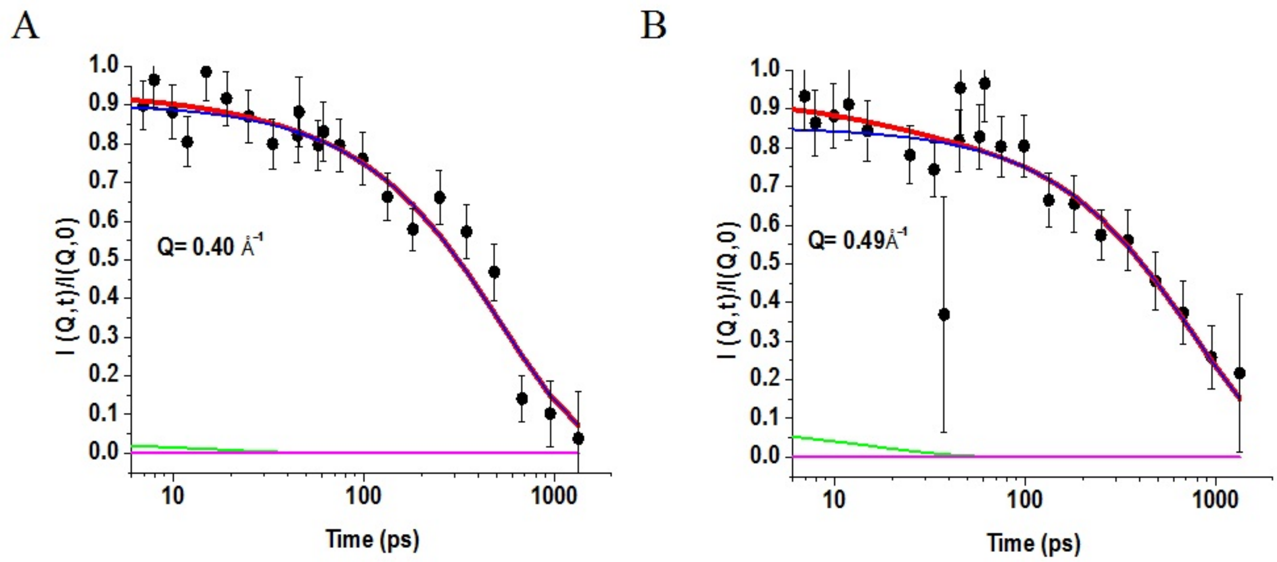

Figure S5. Selected NSE (IN11, ILL, France) spectra of bulk B(d9)MIM-TFSI at 298 K. The red thick line is the fit Eq.S16 and the three dynamical contributions are shown: side-chains (Eq.S4, pink line), local diffusion within an aggregate (Eq.S5, green line) and long-range diffusion of the whole cation (Eq.S8, blue line).

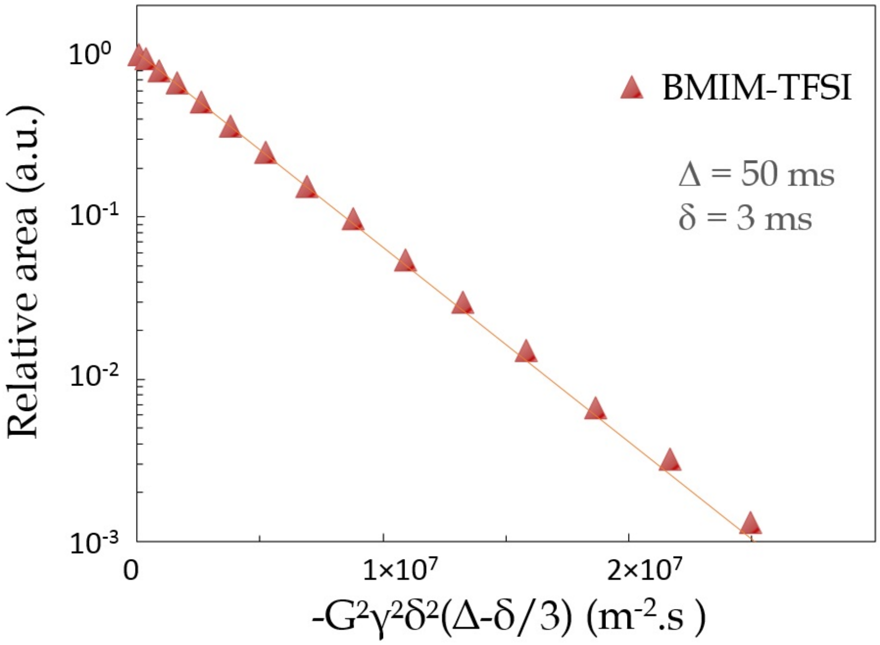

Figure S6. ^{1}H PFG-NMR measurements of the self diffusion coefficient of BMIM-TFSI: $2.7 \pm 0.1 \times 10^{-7}$ cm^2/s. This quantity is measured on length and time scales order of magnitudes larger than the ones accessible by QENS/NSE: (μm,*ms*) *vs* (*nm*, *ns*).

Parameters	BMIM-TFSI
p (Eq.S4)	12/15 = 0.8
$6\sigma_{loc}$ (Å)	11.4 ± 1.2
D_{loc} (cm^2/s)	$4.8 \pm 0.3\ 10^{-5}$
D_{lr} ($cm\ ^2/s$)	$1.6 \pm 0.3\ 10^{-6}$
D_{NMR} ($cm\ ^2/s$)	$2.7 \pm 0.1\ 10^{-7}$
D_{lr}/D_{NMR}	6 ± 1

Table 1. Summary of the QENS,NSE and NMR experimental results.

References

1. Bee, M. *Quasielastic Neutron Scattering, Principles and Applications in Solid State Chemistry, Biology and Materials Science*. (CRC Press, 1988).
2. Burankova, T., Hempelmann, R., Wildes, A. & Embs, J. P. Collective Ion Diffusion and Localized Single Particle Dynamics in Pyridinium-Based Ionic Liquids. *J. Phys. Chem. B* **118**, 14452-14460 (2014).
3. Volino, F., Perrin, J.-C. & Lyonnard, S. Gaussian Model for Localized Translational Motion: Application to Incoherent Neutron Scattering. *J. Phys. Chem. B* **110**, 11217-11223 (2006).
4. Sears, V. F. Theory of cold neutron scattering by homonuclear diatomic liquids: II. Hindered rotation. *Canadian Journal of Physics* **44**, 1299-1311 (1966).
5. Ordikhani Seyedlar, A., Stapf, S. & Mattea, C. Dynamics of the ionic liquid 1-butyl-3-methylimidazolium bis(trifluoromethylsulphonyl)imide studied by nuclear magnetic resonance dispersion and diffusion. *Phys. Chem. Chem. Phys.* **17**, 1653-1659 (2015).
